# Machine learning and clinical epigenetics: a review of challenges for diagnosis and classification

**DOI:** 10.1186/s13148-020-00842-4

**Published:** 2020-04-03

**Authors:** S. Rauschert, K. Raubenheimer, P. E. Melton, R. C. Huang

**Affiliations:** 1 Telethon Kids Institute, University of Western Australia, Nedlands, Perth, Western Australia; 2 School of Medicine, Notre Dame University, Fremantle, Western Australia; 3 Centre for Genetic Origins of Health and Disease, The University of Western Australia and Curtin University, Perth, Western Australia; 4 School of Pharmacy and Biomedical Sciences, Curtin University, Bentley, Western Australia; 5 Menzies Institute for Medical Research, University of Tasmania, Hobart, Tasmania Australia

## Abstract

**Background:**

Machine learning is a sub-field of artificial intelligence, which utilises large data sets to make predictions for future events. Although most algorithms used in machine learning were developed as far back as the 1950s, the advent of *big data* in combination with dramatically increased computing power has spurred renewed interest in this technology over the last two decades.

**Main body:**

Within the medical field, machine learning is promising in the development of assistive clinical tools for detection of e.g. cancers and prediction of disease. Recent advances in deep learning technologies, a sub-discipline of machine learning that requires less user input but more data and processing power, has provided even greater promise in assisting physicians to achieve accurate diagnoses.

Within the fields of genetics and its sub-field epigenetics, both prime examples of complex data, machine learning methods are on the rise, as the field of personalised medicine is aiming for treatment of the individual based on their genetic and epigenetic profiles.

**Conclusion:**

We now have an ever-growing number of reported epigenetic alterations in disease, and this offers a chance to increase sensitivity and specificity of future diagnostics and therapies. Currently, there are limited studies using machine learning applied to epigenetics. They pertain to a wide variety of disease states and have used mostly supervised machine learning methods.

## Background

Clinical epigenetics is a promising field of research. There is evidence that DNA methylation changes at cytosine-phosphate-guanine (CpG) sites are associated with disease development [1–3]. Beyond genetic background, DNA methylation may additionally reflect environmental exposures and could improve diagnostic accuracy and prognostic prediction of certain diseases and be targetable by personalised therapy in the future [4, 5].

The current medical environment is characterised by collection of vast amounts of patient, hospital, and administrative data [6, 7], which makes traditional approaches to investigating these data individually less ideal. Machine learning (ML), however, is able to integrate large and complex data sets [8]. These data sources have the potential to enhance patient care and outcomes. A personalised medicine approach is tightly connected to increases in *omics-data*. For example, DNA sequence databases double in size twice a year [9]. Indeed, the increases in computer processing coupled with the rapid reduction in the cost of genomic sequencing have outpaced the rate of computing hardware advances [10]. Whilst far from a panacea, ML may be a tool to assist physicians in interpreting information-rich clinical data, including those collected in epigenetic studies [11, 12].

This review was guided by the question, “What are the machine learning models that utilize DNA methylation to classify or diagnose disease states?” This review focused on three key aspects within the search strategy, namely, the data science technique, the biomedical technique, and the outcome of interest. The search strategy involved two databases, namely, PubMed and Google Scholar. The search string for the PubMed database was as follows: (‘machine learning’ OR ‘artificial intelligence’) AND (“epigenetic*” OR “DNA methylation”) AND (“classification” OR “diagnosis”). For Google Scholar, the terms machine learning, artificial intelligence, epigenetic, DNA methylation, classification, and diagnosis were utilized. Following the identification of key articles, references in the identified articles were checked to further identify relevant literature (*n* = 1). Once selected, all literature was evaluated for the type of ML utilized, the type of DNA methylation technique used, ML performance measures, validation technique, and the number of samples and number of controls in testing sets and validation sets.

This review is written in the context of the concurrent burgeoning interest for the medical practitioner in potential clinical applications of epigenetics and ML. The first aim of this review is to provide a brief overview of epigenetics, followed by its clinical application potentials. The second aim is to provide a brief summary of the current state of ML and its application to the field of epigenetics and personalised medicine. Finally, section three delves into future directions that may be of value to scientists and physicians looking to harness the power of ML in epigenetics. As the field of ML is likely to find widespread application in clinical practice via diagnostic tools, this review aims to be a brief guide to the current state of ML in epigenetics.

## Epigenetics and its clinical potential

Epigenetics, sometimes described as the study of heritable changes in gene expression that occur without a change in DNA sequence [13], is postulated to be the product of a complex interaction between an individual’s genotype, age, and lifestyle factors such as diet, alcohol consumption, and smoking [14–17]. In 1942, the term “epigenetics” was first coined by Conrad H Waddington [18]. The word is derived from the Greek word “epigenesis”, and initially described the influences of genetic processes on development [18].

Several diseases have been shown to be associated with differential DNA methylation including various cancers, obesity, and cardiovascular disease [19–23]. Broadly, four major categories of epigenetic changes exist: DNA methylation, RNA-centred mechanisms (including non-coding RNAs and microRNAs), histone modifications, and chromatin conformation [24]. Of these, DNA methylation is the most commonly studied epigenetic modification in mammals, particularly methylation of a cytosine molecule adjacent to a guanine molecule [25]. The cytosine-guanine dinucleotide is referred to as a CpG site and these sites often occur in clusters termed CpG islands [26].

One of the most popular methods of measuring genome-wide DNA methylation profiles is through microarrays, chiefly the Illumina HumanMethylation Infinium BeadArray [27]. Each generation of the Illumina technology has been associated with diminishing cost and a larger portion of the genome measured, with the number of CpG sites measured from ~ 27,000 [28] to ~ 450 000 [29] and most recently to ~ 850,000 with the EPIC array [30]. Other techniques, such as pyrosequencing and methyl-sensitive endonuclease restriction, are potentially more accurate than the Illumina HumanMethylation microarray technique, but only suitable for low-throughput studies, as they are also very time-consuming [27]. Therefore, whilst the Illumina microarray has limitations, it is still one of the most widely used DNA methylation techniques in the epigenetic field [27, 31].

A recent review *in Nature Review Genetics* gives a comprehensive overview of the clinical potential of epigenetics [32]. Epigenetics is closely linked to environmental influences and hence potentially better suited to disease diagnosis and treatment than genetics alone [32]. As epigenetics has been shown to play a role in the mediation between early life adverse environments and later life disease onset, it has a potential role for early diagnosis [33]. It has been shown that adverse early life, such as famine [34] or exposure to maternal smoking during pregnancy [15, 35], can program the development of the child mediated on an epigenetic level [36].

However, the biggest successes to date in using epigenetic information as a biomarker have been achieved in oncology, where biomarkers have been approved by the US Food and Drug Administration [37]. One such example is the ^m^SEPT9 biomarker for colorectal cancer, which has been discovered in 2003 and is now a commercialized kit that can diagnose colorectal cancer in blood plasma based on epigenetic markers [37].

To date, ML has yielded limited biomarkers that have made it into current clinical practice. However, it is likely that in the upcoming decades the application of ML to the epigenome [38] will yield many more potential biomarkers and drug targets, particularly because ML is optimized to find meaning in large and complex data sets. In genomics and transcriptomics, ML methods are already used for example in gene set enrichment analysis, to find highly overrepresented pathways [39].

## Overview of machine learning and systematic literature review for machine learning in epigenetics

AI, as part of computer science, uses algorithms to allow computers to perform traditionally ‘human’ executive functions such as problem-solving and decision-making [40]. AI includes fields such as natural language processing, expert system, robotics, and ML [41]. The various biomedical applications of AI fields other than ML is beyond the scope of the current review, and substantial reviews are available elsewhere [40, 42–44]. As previously mentioned, one subdiscipline of AI that shows strong potential in the field of data-driven medical fields is that of ML [11, 45].

**Fig. 1:**
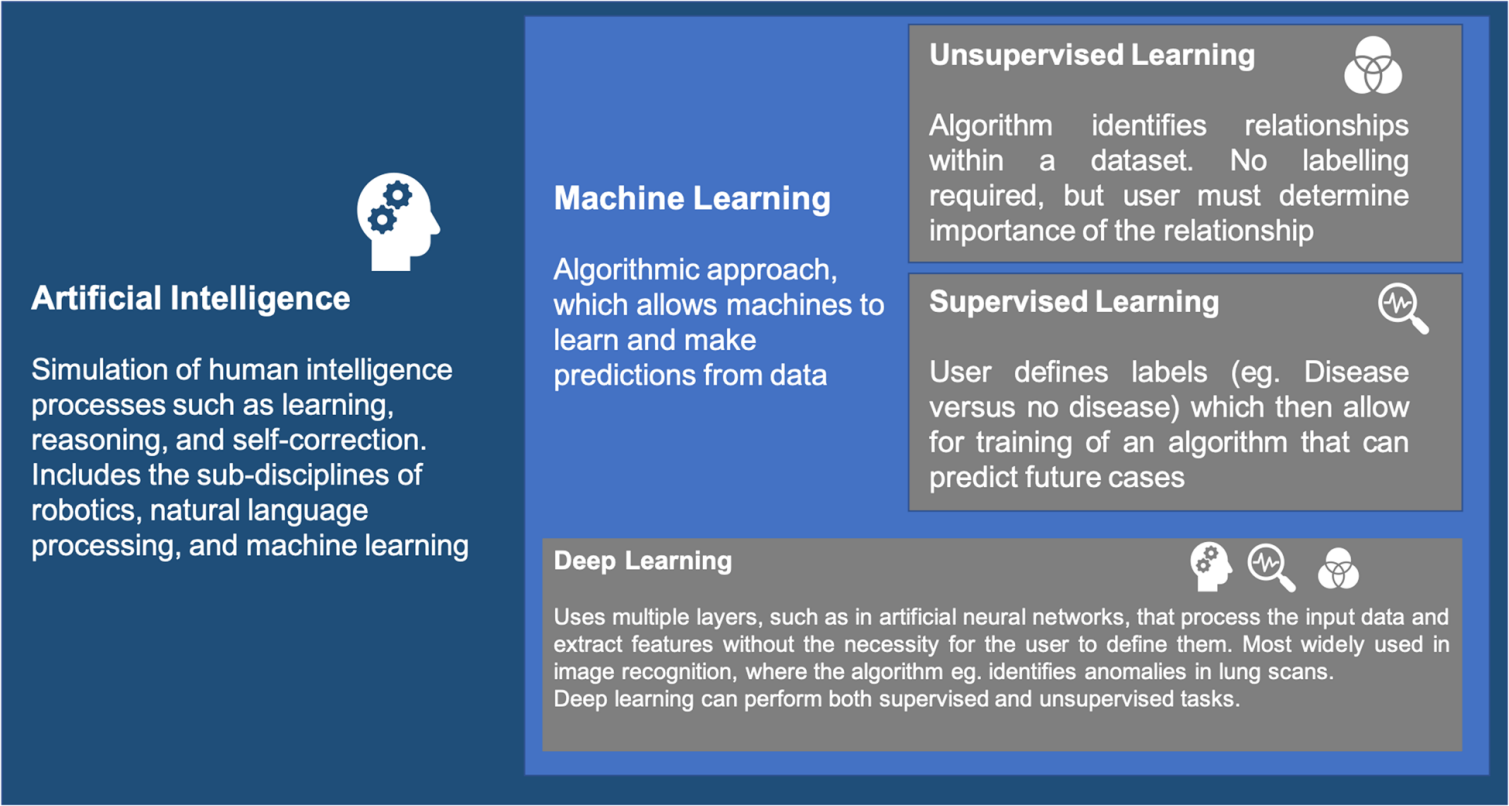
Overview of the field of artificial intelligence and its sub-field machine learning

ML enables computers to learn and make predictions by finding patterns within the data [40]. With increased amounts of data available, ML approaches become more adept at pattern prediction, a factor that makes ML particularly suited to data-rich medical fields like genomics and its sub-field epigenetics. ML algorithms are generally categorised into supervised, unsupervised, and deep learning. A simplified visual representation of the relationship between these fields is presented in Fig. 1.

**Fig. 2:**
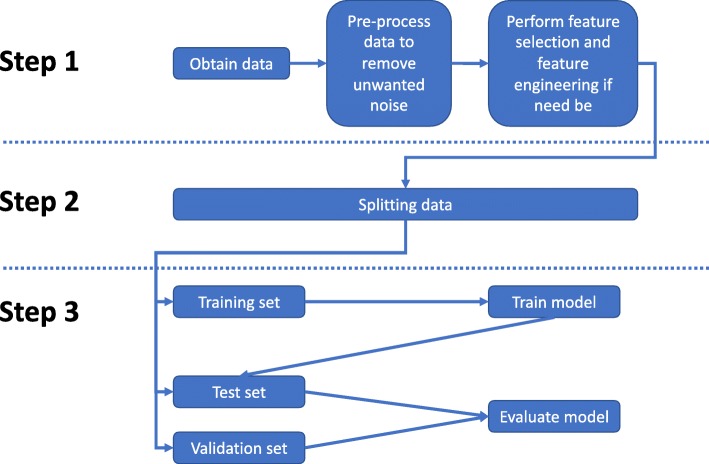
Workflow for applying a machine learning algorithm

Within the field, there are some essential concepts that clinicians ought to be familiar with when considering ML. A simplified approach to steps for developing and applying an ML algorithm is outlined in Fig. 2. A suggested processing pipeline is to split the available data into three sub data sets: a training data set, where the selected algorithm is optimised and the parameters are evaluated, a test data set, where the performance of the trained algorithm is evaluated, and a validation data set, which ideally comes from a different source than the training and test data set. This last step, the validation, is not always possible due to unavailability of data but allows for a more robust estimation of the algorithm performance beyond the training data set. A good alternative for this is k-fold cross-validation. This means, during the training process, the data is randomly split into k training and test sets, which allows for a good approximation of the external validity of the model [46]. Common performance measures employed in classification tasks that use balanced data sets for training are accuracy, sensitivity, specificity, and precision [47, 48]. For imbalanced data sets (low number of cases versus controls), more robust performance evaluators that take into account class distribution are more appropriate, for example, *F*_*1*_-score, area under the curve (AUC), and Cohen’s Kappa [47–49].

### Supervised learning

Supervised learning is a subset of ML where labels to a dataset are known, for example, cancer patients versus healthy controls, which is subsequently used to train an algorithm that can make predictions about the health outcome on unseen data, without knowing the disease status [11, 40]. This form of ML is reliant on user input to categorise the different instances in the learning process. Supervised learning algorithms have been effectively utilised in classification and prediction tasks [50]. Commonly used algorithms within this category of ML include linear or logistic regression, support vector machine, random forest algorithms, and least absolute shrinkage and selection operator regression (LASSO) [40]. Briefly, support vector machine is based on the idea that by transforming the data, eventually it will be possible to separate classes by a hyperplane, which in the two-dimensional space is a simple line [51]. The points nearest to this hyperplane are called support vectors and are essential for the classification [51]. A Random Forest algorithm is a decision tree-based model, that builds up a multitude of decision trees of differing depth [52]. Further, for every tree, a random subset of the data set is utilised and at every split in the decision tree, a random subset of the features is used. This makes every decision tree in the forest highly uncorrelated to the next and the final predictor, which is an average of the whole ensemble of trees, will be highly unbiased [52]. Finally, LASSO is a logistic regression based model that also performs feature selection, meaning the most important variables for prediction are selected from the data set via a so-called penalization model that weighs the features depending on their effect [40]. For further information and details on the algorithms, please refer to the original publications referenced here [40, 51, 52].

Examples of supervised learning using epigenetic data include classification of metastatic brain tumours, prostate cancer, coronary heart disease, neurodevelopmental syndromes, and central nervous system tumours [53–57]. This review focuses on supervised learning, as this is mostly used when trying to develop a diagnostic test to assist clinicians in the diagnostic process (examples: Tabl 1).

Whilst supervised learning provides a robust method by which to classify diseases versus healthy individuals, there are inherent limitations. Firstly, supervised learning usually requires user input in order to define training classes (or classify the disease and healthy patients) to develop a model [40]. Secondly, since ML algorithms are sensitive to the quality of the data, it is essential that they be correctly labelled [40]. If the training data has examples that are incorrectly labelled, the supervised learning classifier will make incorrect predictions [40]. Finally, supervised learning is susceptible to ‘over-fitting’—the tendency to work very well on the training data but having limited performance on other external data sets [58]. Despite these limitations, supervised learning is one of the most widely used ML techniques in classification and prediction in epigenetics (Table 1).

**Table 1:** Brief overview of some of the most frequently used performance measures for machine learning models

Performance metric	Interpretation
*Accuracy*	Brief definition: Accuracy is a classifier that works best on balanced data sets. It is a measure that informs about the correct classifications out of all classifications. It can have values from 0 - 100 % Example: If we are dealing with a binary classification, e.g. cancer versus healthy, and we have 20 patients with cancer and 80 healthy controls, a model accuracy of 80% would mean that the model classified every subject into the majority class (healthy) and is completely unable to classify cancer patients, although the accuracy indicates a good performance.
*Sensitivity*	Brief definition: The sensitivity is the true positive rate of a test. This means, how many subjects with a disease are actually identified as having the disease by the test. The values range from 0 to 100%. Example: Let us say we have a epigenetic test, that claims to identify the presence of a specific type of cancer. When evaluating the test, it was able to identify 30 out of 60 cancer patients correctly. The sensitivity of this test would then be 50% (30/60)
*Specificity*	Brief definition: The specificity is the true negative rate of a test. In other words, it represents the proportion of people without the disease, that will have a negative result. Just like for sensitivity, the values range from 0- 100% Example: We assume we are dealing with the same diagnostic test for cancer as in the explanation of sensitivity. Out of 90 healthy subjects, 70 had a negative diagnosis. This means the specificity of the test is 78% (70/90)
*Precision*	Brief definition: Precision is a measure that tells us out of all predicted cases, how many are actual cases. Possible values range from 0 to 1. Example: In the cancer example, how many predicted cancer cases are actual cancer cases.
*Recall*	Brief definition: Recall is a measure that informs us how many cases we were able to identify as cases. The value range is 0 to 1. Example: Out of all the cancer patients, how many was the predictive model able to identify as cancer patients?
*F1-Score*	Brief definition: The F1-score is the harmonic mean between precision and recall. In this case, we aim for both high recall and high precision, meaning we want to be able to identify a large amount of cases and we also want to be sure that the majority of predicted cases are actual cases. The F1-score ranges from 0 to 1, where 0 is the worst performance. Example: If we have a near-perfect precision and recall, meaning we ate able to classify a large amount of the cancer patients as cancer patients (recall) and we are sure that our prediction is correct (precision), the harmonic mean between the two of them for a good model would be ~ 0.9.
*Area under the receiver operator curve (ROC AUC)*	Brief definition: The area under the receiver operator curve is a measure of how sensitive and specific a test performs. In a graphical representation, the *x*-axis depicts the negative predictions and the *y*-axis the positive predictions. If a test performs bad in terms of sensitivity and specificity, the area under the curve would be 0.5, which means it is not better than tossing a coin.

Another class type of algorithm that can be used in supervised ML is deep learning. Deep learning algorithms are capable of processing high volume, high-dimensionality data—data with a high number of variable input sources—and identifying complex patterns [59]. For epigenetics, deep learning provides an enticing avenue to explore. Common deep learning techniques include artificial neural networks and convolutional neural networks [59, 60]. Historically, deep learning is considered one of the more computationally expensive types of AI, requiring large amounts of computing power in order to be effective [59]. The advances of computing power and high-speed internet in the last half a decade has led to efficient and effective use of deep learning, particularly through web-based (super-)computing services such as Amazon Web Services, Google’s Cloud service, and Microsoft Azure.

Perhaps the most problematic issue with deep learning is the inability to identify precisely how the algorithm has determined the outcome, known colloquially as ‘black-boxing’ [61]. Black-boxing is an especially significant limitation in the medical context due to the implications on patient safety and ability to prove clinical reasoning [61, 62].

### Unsupervised learning

In contrast to supervised learning, unsupervised learning does not require labels in order to work [40, 63]. However, whilst unsupervised algorithms provide strength of correlation between individual variables within a data set, they are unable to assign the potential biological relevance and/or plausibility of these patterns of correlation [40, 63]. Therefore, human input is required to assess the biological plausibility and the salience of any associated clusters identified by the algorithm [40, 63]. Common problems that unsupervised learning has been used for include clustering and association tasks [40]. Clustering, as the name suggests, clusters data points according to inherent groupings in the data. Common methods used in unsupervised learning include *k*-means clustering and hierarchical clustering, principle component analysis, and partial least squares discriminant analysis [64, 65]. The latter two methods are often utilised in dimensionality reduction, or the removal of random input variables to increase the performance of a model [66].

Within an epigenetic context, unsupervised learning can be used to detect DNA methylation patterns between diseased and non-diseased individuals, for example, between breast cancer brain metastases subtypes [38, 57]. Unsupervised learning algorithms are especially useful to detect patterns in data sets that have large amounts of data points, such as those in microarray and *omics* data sets [66, 67].

The main limitation of unsupervised learning is that the algorithms do not provide insight into the importance or relevance of clustering and/or associations [68]. The concept of ‘correlation does not mean causation’ is especially relevant to unsupervised ML. Due to the inability of unsupervised ML algorithms to prescribe meaning to associations, caution should be exercised when interpreting any associations identified by an unsupervised ML algorithm, as they may be data artefacts as opposed to true biological effects. Furthermore, unsupervised learning is sensitive to noise within the data [40]. If there is a large amount of irrelevant data within a data set, an unsupervised learning algorithm may cluster points erroneously. Therefore, data used for unsupervised learning must be carefully pre-processed to ensure it is of high quality prior to analysis. Deep learning approaches can also be used for unsupervised tasks. An example of a clinical application is a deep learning model that was trained on unlabelled mammography images to identify breast density scores which showed a very strong positive relationship with manual scores, predictive of breast cancer [69].

### Epigenetics and machine learning: existing literature

Overall, 16 studies were identified that utilised ML to diagnose or classify diseases [39, 54–58, 71–80).

There was extensive heterogeneity in the disease outcomes, types of algorithms, performance measures, validation methods, and sample sizes between studies. Table 1 summarises the studies that have investigated the use of ML for diagnosis or classification in various cancers (*n* = 10), cerebral palsy (*n* = 1), neurodevelopmental syndromes (*n* = 1), coronary artery disease (*n* = 1), and BAFopathies (*n* = 1; disruption of the *BRG1*/*BRM*-associated factor (BAF) complex has been linked to several neurodevelopmental syndromes, commonly referred to as BAFopathies). A special case where the two identified deep learning approaches, DeepCpG and DeepMethyl, as they both predicted methylation status in the genome rather than a disease status [70, 71] (Table 2).

**Table 2:** Overview of the literature on machine learning and clinical epigenetics, including data type, machine learning method used, sample size, and performance measures.

Disease	ML method	Sample size	Epigenetic data type	Performance	Validation method	Authors
Metastatic brain tumours	Random forest	1860 165 patients	Infinium HumanMethylation 450K	AUC for type GBM-A = 0.87 BM-C = 0.82 BM-C–GBM-A = 0.92 AUC for site of origin LuCa, BrCa, Melan = 0.99	Bootstrap	Orozco, 2018 [57]
Cerebral palsy	Non-metric multidimensional scaling Linear discriminant analysis Random forest	22 CP patients 21 controls	Methyl-sensitive restriction endonuclease (MSRE)	Accuracy = 73% Sensitivity = 100% Specificity = 40% AUC = 0.691	Bootstrap 20-fold cross-validation	Crowgey, 2018 [38]
Prostate cancer	Least absolute shrinkage and selection operator	234 PrCa 76 controls	Infinium HumanMethylation 450K	Training set 100% accuracy, sensitivity, specificity, AUC Validation set Sensitivity = 96% Specificity = 98% Accuracy = 97% AUC =98%	None reported	Aref-Eshghi, 2018 [54]
Central nervous system tumours	Random forest	2801 (91 different classes)	Infinium HumanMethylation 450K Infinium HumanMethylation EPIC Whole Genome Bisulphite Sequencing	Cross-validation error rate (raw) = 4.89% Cross-validation error rate (calibrated) = 4.28% AUC = 0.99 8 methylation class error rate = 1.14% Multiclass approach: Sensitivity = 0.989 Specificity = 0.999 Classification concordant with pathology on validation set = 76%	3-fold, nested cross-validation	Capper, 2018 [55]
Neurodevelopmental syndromes	Support vector machine	285 cases across 14 syndromes 650 controls	Infinium HumanMethylation 450K + EPIC	Accuracy = 99.6% Sensitivity = 100% Specificity = 100%	10-fold cross-validation	Aref-Eshghi, 2018 [53]
Coronary heart disease	Random forest	1545 173 with coronary heart disease	Infinium HumanMethylation 450K	Accuracy = 78% Sensitivity = 0.75 Specificity = 0.80	10-fold cross-validation	Dogan, 2017 [56]
BAFopathies	Support vector machine	Cases *n* = 29 (CSS1 = 14; CSS3 = 5; CSS4 = 2; NCBRS = 7) Controls 156 (CSS1 = 84; CSS3 = 30; CSS4 = 0; NCBRS = 42)	Infinium HumanMethylation 450K + EPIC	Testing set Accuracy = 98.8%	10-fold cross-validation	Aref-Eshghi, 2018 [72]
Lung cancer	Multi-class support vector machine	Training set LADC = 126 SQCLC = 134 SCL = 28 Test set LADC = 452 SQCLC = 359	Infinium HumanMethylation 27k (training) Infinium HumanMethylation 450K (independent)	Training set Accuracy = 86.54% ± 2.2 Precision = 66.79% ± 1.9 Recall = 84.37% ± 2.5 *F*-score = 74.55% ± 2.2 Independent sets Accuracy = 84.6% Precision = 85.94% Recall = 85.52% *F*-score = 85.04%	Leave-one-out cross-validation	Cai, 2015 [73]
Cancers	Support vector machine	Comparisons between Male = 7, female = 14 T-ALL/B_ALL = 17 Healthy T/B cells = 13 AML = 8 BPH = 10 Prostate carcinoma = 10 Healthy kidney = 9 Kidney carcinoma = 9 Prostate = 20 Kidney = 18	Bisulphite Sequencing (GenePix4000)	Accuracy Male vs female = 91% T/B cells vs ALL = 94% ALL vs AML = 94% Kidney vs kidney carcinoma = 92% Prostate vs kidney = 92%	50-fold cross-validation	Adorjan, 2002 [74]
Breast cancer	Random forest	543 TCGA, gene expression, and methylation	Infinium HumanMethylation 450K	Bootstrap error = 20% Average AUC = 88%	.632 bootstrap error	List, 2014 [75]
Lung cancer	Random forest support vecor machine linear regression naïve Bayes	50	Infinium HumanMethylation 450K (+ CHIP-Seq from ENCODE)	Training set AUC = 86.4% Test set AUC = 83.6%	10-fold cross-validation	Li, 2015 [76]
CLL subtypes	Support vector machine	Training set 211 Validation set 97	Bisulphite pyrosequencing	Not reported. Authors just state the prediction was accurate.	.632 bootstrap error	Queiros, 2015 [77]
CLL subtypes	SVM	135	Bisulphite pyrosequencing (PyroMark)	No testing of algorithm	NA	Bhoi, 2016 [78]
Various cancers	One class logistic regression	12000 (33 cancers)	Infinium HumanMethylation 450K	None reported	None	Malta, 2018 [79]
Prediction of methylation in leukemia and healthy cells	Deep learning via deep methyl using stacked denoising autoencoder	Two cell lines: GM12878: B-lymphocyte cell line from a female K562:immortalised cell line from a female patient with chronic myelogenous leukemia	Reduced representation bisulfite sequencing (RRBS)	Accuracy GM12878: 84.82% for unknown neighbouring regions 89.7% blinded K562 72.01% for unknown neighbouring regions 88.6% blinded	leave-one-out cross-validation	Wang, 2016 [70]
Prediction of methylation status of single cells	Convolutional neural network	18 serum-cultured mouse embryonic stem cells 25 human hepatocellular carcinoma cells, 6 human hepatoblastoma-derived cells 6 mESCs	Single-cell bisulphite sequencing single-cell reduced representation bisulphite sequencing	Based on additional file 2 of the publication: Mean/sd accuracy: 87.9%/0.09% Mean/sd AUC: 0.87/0.08 Mean/sd F1: 0.67/0.21	Holdout validation	Angermueller, 2017 [71]

The types of algorithms used have all been supervised learning, including support vector machines (*n* = 7), random forest (*n* = 7), LASSO regression (*n* = 1), non-metric multidimensional scaling (*n* = 1), logistic regression (*n* = 1), convolutional neural network (*n* = 1), and stacked denoising autoencoder (*n* = 1). Of note, some research used multiple models.

The types of epigenetic data include microarray techniques (*n* = 11), bisulphite sequencing (*n* = 3), and methyl-sensitive restricted endonuclease (*n* =1). Of these collection methods, most studies used one type of DNA methylation technique only (*n* = 9), whilst others combined measurement techniques, meaning Infinium HumanMethylation 450K and EPIC or CHIP-Seq from The Encyclopedia of DNA Elements (ENCODE) (*n* = 5).

From the selected publication, it appears that the two most popular methods were support vector machine and random forest. Based on the approaches identified, it seems the most successful combination is 10-fold cross-validation with either a random forest or support vector machine for array-based methods and deep learning-based models for prediction of the methylation status of the DNA.

Epigenetic data have traits that make it amenable to ML. Firstly, DNA methylation is usually both chemically and biologically stable over time [5]. Consequently, the measurement of DNA methylation allows for a reliable measure of the chemical composition of the epigenome at any given point in time. Secondly, large-scale, data-rich repositories such as The Cancer Genome Atlas (TCGA), ENCODE, and the BLUEPRINT consortium provide large amounts of samples to employ comprehensive, high-throughput statistical analyses of differentially methylated regions with biological relevance [80–82]. These repositories may provide for the training data for a ML algorithm, or an independent test set in order to determine the ML algorithm’s external validity and subsequent clinical utility [81, 83]. Since ML algorithms require large amounts of data to make accurate predictions, the establishment of these databanks is a significant milestone in the utility of AI in epigenetics. Finally, most datasets consist of DNA methylation profiles derived from peripheral blood, meaning that patients will only be required to provide a small blood sample. It should be noted that DNA methylation profiles are tissue-specific, and that the use of peripheral blood as a measure of DNA methylation may be less useful in diseases such as certain cancers [84], with more clinical utility in diseases like obesity [85, 86].

## Challenges and future perspectives

Whilst there are advantages to combining epigenetics with ML to assist clinicians in the diagnostic process, there are significant challenges that must be addressed. First, very large datasets, requiring cross-jurisdiction collaboration are needed, especially if the diseases that need prediction are rare. This problem occurs 2-fold in epigenetic data: initially with the patient to healthy control ratio (with many datasets containing many more controls as compared to disease cases) and secondly within the individual methylomes, where there is a higher proportion of sections in the DNA that are densely methylated, referred to as differentially methylated regions (DMR), compared to the number of non-DMR sites [12, 87]. Second, most epigenetic data sets have more variables than samples, making it difficult for many ML algorithms to function effectively [88]. A potential solution is to collect more data, something that collaborative data repositories are providing. Concurrent, careful consideration of the type of algorithm and suitable performance measures of the prediction should be made to prevent erroneous data interpretations.

Third, not all associations in a DNA methylation dataset are linear. Several CpGs may be linked to the same gene which may influence other portions of the methylome and transcriptome, which has particularly been identified as an issue in gene set enrichment analysis [89, 90]. Additionally, the Illumina HumanMethylation450 array only covers 2% of all CpG sites in the methylome [27]. These challenges must be recognised before the full clinical potential of epigenetics is realised.

Fourth, for proper development, improvement and testing of novel machine learning approaches, it will be crucial to increase efforts to make large epigenetic datasets publicly available. This should include the raw data of different platforms, so research can be conducted into the effect of different normalisation methods on ML model performance and assessing which models work best for array-based and bisulphite sequencing-based data formats. One of the largest efforts in providing access to sequencing data is provided by The National Center for Biotechnology Information (NCBI). This includes databases such as the sequencing read archive (SRA) that are invaluable for research into new computational methods [91]. The SRA is operated by the International Nucleotide Sequence Database Collaboration (INSDC) and was initially started to publicly deposit sequencing reads [91]. Currently, more and more funding bodies and scientific journals request a deposition of experiment data in the SRA, which is not only beneficial for reproducibility of research, but also for efforts into the development of new analytical tools. Resources such as SRA made it possible to develop sequencing analysis tools such as Magic-BLAST (Basic Local Alignment Search Tool), which allows to align sequencing reads to a reference genome based on a sequencing database [92].

In an epigenetic context, deep learning has been used to classify genetic mutations in gliomas and prediction of single-cell DNA methylation status [71, 93]. Whilst still in its infancy, applications of deep learning to classification tasks using DNA methylation data may have benefits over traditional ML.

Another challenge for the field of ML is prediction bias. Several cases in facial recognition, especially relevant to deep learning because of their black box character, have shown that the predictive models are biased towards populations of European ancestry [94]. Therefore, the challenge of getting representative datasets that do not exacerbate existing health differences for disadvantaged populations is one of the biggest challenges that the ML community needs to address [95].

## Conclusion

As an in-depth introduction to epigenetics and ML was out of the scope of this review, we aimed to give an overview of epigenetics and the potential of ML in clinical applications. The interested reader may refer to the cited literature on the different topics of epigenetics and machine learning.

ML is starting to find patterns in ever-growing genetic and epigenetic data sets that relate to the development of diseases. Although very accurate, deep learning methods will need to undergo further research to define what is going on within the “black box”, before clinicians can confidently make informed decisions whilst utilising such tools. In the meantime, interpretable ML algorithms are likely to be on the horizon with the potential to assist in more confident diagnoses. Whilst ML is sometimes depicted in the media and literature as a threat to the clinician’s profession and autonomy, clinicians should perhaps view its application as an assistive tool. ML can be used, just like evolving technologies across the ages (from the stethoscope, to X-Rays, to MRIs) as providing adjunctive information; it is a matter of being properly informed about limitations of the method of algorithm development and understanding where and to whom it is appropriate to apply.

## Data Availability

Not applicable

## Abbreviations

AI: Artificial intelligence
AUC: Area under the curve
CpG: Cytosine phosphate guanine
DMR: Differentially methylated region
ENCODE: the Encyclopedia of DNA Elements
LASSO: Least absolute shrinkage and selection operator
ML: Machine learning
TCGA: The Cancer Genome Atlas
